# Longitudinal impact of gender-affirming endocrine intervention on the mental health and well-being of transgender youths: preliminary results

**DOI:** 10.1186/s13633-020-00078-2

**Published:** 2020-04-30

**Authors:** Christal Achille, Tenille Taggart, Nicholas R. Eaton, Jennifer Osipoff, Kimberly Tafuri, Andrew Lane, Thomas A. Wilson

**Affiliations:** 1 Division of Pediatric Endocrinology, Stony Brook Children’s Hospital, Stony Brook University, Stony Brook, NY 11794-8111 USA; 2 Department of Psychology, Stony Brook University, Stony Brook, NY 11794 USA

**Keywords:** Transgender, Transgender management, Transgender youth, Depression, Suicide, Suicidal ideation, Quality of life, GnRH analogue, Puberty suppression, Puberty, Testosterone, Estrogen, Cross sex hormone

## Abstract

**Background/aims:**

Transgender youths experience high rates of depression and suicidal ideation compared to cisgender peers. Previous studies indicate that endocrine and/or surgical interventions are associated with improvements to mental health in adult transgender individuals. We examined the associations of endocrine intervention (puberty suppression and/or cross sex hormone therapy) with depression and quality of life scores over time in transgender youths.

**Methods:**

At approximately 6-month intervals, participants completed depression and quality of life questionnaires while participating in endocrine intervention. Multiple linear regression and residualized change scores were used to compare outcomes.

**Results:**

Between 2013 and 2018, 50 participants (mean age 16.2 + 2.2 yr) who were naïve to endocrine intervention completed 3 waves of questionnaires. Mean depression scores and suicidal ideation decreased over time while mean quality of life scores improved over time. When controlling for psychiatric medications and engagement in counseling, regression analysis suggested improvement with endocrine intervention. This reached significance in male-to-female participants.

**Conclusion:**

Endocrine intervention may improve mental health in transgender youths in the US. This effect was observed in both male-to-female and female-to-male youths, but appears stronger in the former.

## Introduction

Transgender individuals have a gender identity that differs from the sex assigned at birth [1]. These individuals have a high prevalence of body image dysphoria, depression and suicidal ideation [2]. Studies in adults have shown improvement in psychological function in adulthood from endocrine and/or surgical interventions. Specifically, studies have indicated a positive impact of cross sex steroid therapy on depression scores and quality of life in the adult transgender population [3]. Guidelines for endocrine intervention in transgender youth have existed for the past decade in the United States and longer internationally. These guidelines include suppression of puberty to provide more time before cross sex steroid therapy is introduced [4, 5]. Two studies have examined the impact of this strategy on depression and quality of life in youths. De Vries et al. demonstrated no improvement of gender dysphoria after puberty suppression alone but did report improvement only after both cross sex steroid therapy and gender confirmation surgery was complete in transgender individuals from the Netherlands [6]. These authors did not report findings after cross sex steroid therapy alone but before surgery. In the UK, Costa found that GnRH agonist suppression of puberty improved psychological functioning in transgender youth [7]. In the United States, there are few data concerning the impact of endocrine intervention on psychological function in transgender youth. Therefore, we conducted a longitudinal assessment of psychological wellbeing and quality of life in children and adolescents who have sought endocrine intervention to help with gender dysphoria. Herein, we report preliminary results of this ongoing study.

## Objective

The aim of this study is to examine the impact that endocrine intervention [suppression of endogenous pubertal hormones utilizing GnRH agonists/anti-androgens/suppressors of menstruation (AKA “pubertal suppression”), or addition of cross-sex hormones] has on depression and quality of life scales of transgender youths as reported by the youths themselves over time.

## Methods

### Participants and procedure

This is a single center study approved by Stony Brook University IRB for children, adolescents and young adults aged 9–25 years. Subjects referred to the Pediatric Endocrine Department for gender dysphoria were approached to participate. Although we do not have exact numbers, the vast majority of eligible subjects agreed to take part in the study. Minor participants signed assent and participants over 18 years of age and parents of those less than 18 yr. of age signed consent to participate. Individuals with sex chromosome abnormalities and disorders of sexual differentiation were excluded from the study. At approximately 6-month intervals, participants completed the following validated assessments of mental health: The Center for Epidemiologic Studies Depression Scale (CESD-R) [8], The Patient Health Questionnaire Modified for Teens (PHQ-9_Modified for Teens) [9], Quality of Life Enjoyment and Satisfaction Questionnaire (QLES-Q-SF) [10]. Most subjects were followed by mental health professionals. Those that were not were encouraged to see a mental health professional.

### Psychological measures

The CESD-R score is calculated as a sum of 20 questions, ranging from 0 (for those who say “not at all or less than one day” to all 20 questions) to a maximum score of 60 (for those who say “5–7 days” and/or “nearly every day for 2 weeks” for all 20 questions). A total CESD-R score less than 16 implies no clinical depression [8, 11]. The PHQ-9 consists of 9 questions describing symptoms of depression each rated 0 to 3 with the sum indicating level of depression: minimal 0–4, mild 5–9, moderate 10–14, moderately severe 15–19, severe 20–27. This questionnaire also asks the participants four additional questions relating to suicidal ideology and difficulty dealing with problems of life [9]. The QLES-Q-SF consists of 15 questions rating quality of life on a scale of 1–5 with 1 being poor and 5 being very good [10]. It was used rather than the Pediatric Quality of Life and Enjoyment Scale (PQLES-SF), which is based on QLES-Q-SF, because of the overlap in age inclusion of older adolescents and young adults and the intention of continuing the study into adulthood. Transyouths in the study were also asked if they were participating in psychological counseling and/or on psychiatric medication. ADHD medications were not included as psychiatric medication for this analysis.

### Endocrine interventions

Endocrine interventions were introduced in accordance with the Endocrine Society and the WPATH guidelines [4, 5]. In our study, GnRH agonist and/or antiandrogens were used for male to female (MTF) participants, and suppression of menstruation (either GnRH agonist or Medroxyprogesterone) for female to male (FTM) participants. Collectively, these interventions were labeled “Puberty Suppression”. Once eligible as determined by mental health consultants, youths, parents and according to guidelines, cross sex hormones were prescribed, either testosterone for FTM or estrogen for MTF participants.

### Statistical analysis

Regression analysis was used to examine the association of various treatments with outcomes experienced by transgender youths over time. Linear multiple regression was used for continuous outcomes, and multiple logistic regression was used for dichotomous outcomes. For continuous outcomes, residualized change scores were used to compare change at outcome relative to levels at baseline. This approach thus allowed us to control for the dependent variable’s level at baseline for each participant and to examine how endocrine intervention predicted change in the dependent variable over and above predicted outcome level relative to the level at baseline. Regression analyses also controlled for psychiatric medication and engagement in psychotherapy.

## Results

Between December 2013 to December 2018, 116 participants entered the study. Ninety-five were naive to any endocrine intervention. Of those 95 participants, 50 completed 3 waves of questionnaires and these individuals compose the analytic sample in this report. Baseline data for this population are shown in Table 1. At wave one, none of the 50 participants were on endocrine intervention. By wave 3, 47 participants had some type of endocrine intervention (Table 2).

**Table 1:** Baseline characteristics at Wave 1

	Total	Female to Male	Male to Female
Number of participants	50	33	17
Age in Years (SD)	16.2 (2.2)	16.6 (2.5)	15.5 (1.6)
%Depressed in past year (n)	64% (32)	60.6% (20)	70.6% (12)
% Suicidal (n)	10% (5)	9.1% (3)	11.8% (2)
% In Counseling (n)	90% (45)	87.9% (29)	94.1% (16)
% On Psych Medication (n)	34% (17)	36.4% (12)	29.4% (5)

**Table 2:** Endocrine interventions at wave 3

Type of Intervention	% of Total (n)	% of FTM (n)	% of MTF (n)
None	6% (3)	3% (1)	12% (2)
Puberty Blocker	46% (23)	24% (8)	88% (15)
Cross Sex Hormone	70% (35)	85% (28)	41% (7)
Both	22% (11)	12% (4)	41% (7)

### Mean changes over time

**Fig. 1:**
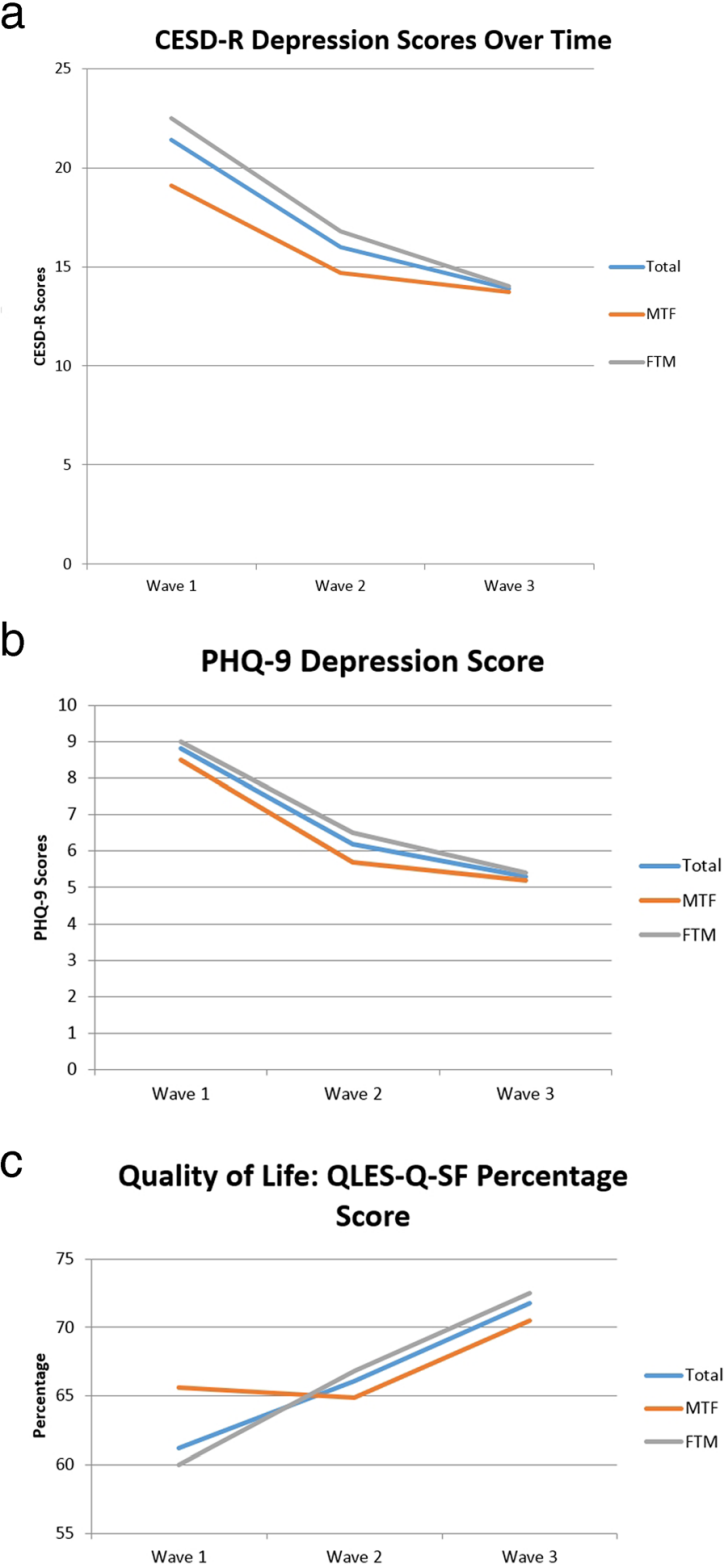
Mean scores over time: **a** CESD-R. **b** PHQ-9. **c** QLES-Q-SF. Abbreviations: GnRH: Gonadotropin releasing hormone; CESD-R: Center for Epidemiologic Studies Depression Scale; PHQ-9: Patient Health Questionnaire Modifed for Teens; QLES-Q-SF: Quality of Life Enjoyment and Satisfaction Questionnaire; WPATH; World Professional Association for Transgender Health; MTF: male to female; FTM: Female to male

Mean baseline CESD-R score was 21.4 and decreased to 13.9 by wave 3 (*t*(48) = 3.996, *p* < 0.001, Fig. 1a). A score less than 16 implies no clinical depression. Mean depression scores by the PHQ-9 decreased over time as well (*t*(49) = 3.753, *p* < 0.001, Fig. 1b), while quality of life scores improved (Fig. 1c) but did not reach statistical significance (*t*(48) = − 1.758, *p* = .085, Fig. 1c). Suicidal ideation decreased over time across all groups at wave 3 relative to baseline (Table 3). Thus, by all measures, depression and quality of life improved to some degree over time. Both gender subgroups demonstrated similar trends.

**Table 3:** Suicidal ideation

Suicidal Ideation Percentage: Wave 1 vs Wave 3		
	% at Wave 1 (n)	% at Wave 3 (n)
Total	10% (5)	6% (3)
MTF	11.8% (2)	5.9% (1)
FTM	9.1% (3)	6.1% (2)

### Regression analysis

We conducted a series of regression analyses to investigate preliminary trends in the data when controlled for reported psychiatric medications and engagement in counselling. Results are given in Table 4. Given our modest sample size, particularly when stratified by gender, most predictors did not reach statistical significance. This being said, effect sizes (*R*^2^) values were notably large in many models. In MTF participants, only puberty suppression reached a significance level of *p* < .05 in the CESD-R. However, associations with PHQ9 and QLES-Q-SF scores approached significance. For FTM participants, only cross sex hormone therapy approached statistical significance for quality of life improvement (*p* = 0.08).

**Table 4:** Regression results when controlled for engagement in counselling and psychiatric medications

		MTF			FTM		
Survey	Intervention	b	***p***	R^**2**^	b	***p***	R^**2**^
CESD-R	Puberty Suppression	−2.41	0.008	0.52	−0.02	0.95	0.09
	Cross Sex Hormone	−0.56	0.27	0.21	− 0.43	0.43	0.11
PHQ-9	Puberty Suppression	−1.89	0.07	0.28	−0.16	0.68	0.04
	Cross Sex Hormone	−0.92	0.07	0.29	−0.23	0.67	0.04
QOL	Puberty Suppression	1.26	0.21	0.13	0.71	0.86	0.01
	Cross Sex Hormone	0.87	0.06	0.08	0.93	0.08	0.11

Model *R*^2^ values ranged between small to large, even in models where the hormonal intervention’s prediction of the outcome did not reach statistical significance. It is potentially noteworthy that effect sizes for endocrine interventions were notably larger for MTF than FTM participants in almost every analysis. Regression models for suicidal thoughts were not estimable due to the low frequency of endorsement and small cell sizes across gender.

## Discussion

Cross-sex hormones and their effect on depression and quality of life has been extensively studied in adults. A meta-analysis by Costa and Colinza reported a reduction in anxiety and depression and improvement in quality of life with positive effect on personality and mood among transgender adults receiving cross-sex hormones therapy [3]. A 2006 cross-sectional study in California looked at adult FTM transgender participants on cross-sex hormone therapy and their quality of life. Participants who received testosterone therapy reported statistically significant higher quality of life than those who had not received hormonal therapy [12].

Adolescence is a particularly difficult time for transgender persons who experience the development of secondary sexual characteristics that are incongruous with their gender identity, and is associated with a high prevalence of depression and suicidal thoughts and gestures. Previous research has shown benefit to transgender youth in the Netherlands after cross sex steroid therapy AND gender confirmation surgery and in the UK after pubertal suppression alone [6, 7]. Our results extend these findings to transgender youths in the USA and apply prior to surgery.

Our results suggest that endocrine intervention is associated with improved mental health among transgender youth. This effect was observed in both MTF and FTM participants but appeared to be stronger in MTF. We speculate that this could be due to the following possibilities: 1. Testosterone has profound effects on appearance. MTF participants may have experienced relief when serum testosterone concentrations are suppressed or antagonized; 2. The effects of testosterone in FTM transgender persons takes 6 to 12 months to become apparent and is not fully apparent until several years of exposure. Our study only extended for the first 12 months of endocrine intervention.

## Limitations and future directions

This is an ongoing study with preliminary results only presented herein. The numbers are too small to parse out the effects of pubertal suppression versus cross sex hormone therapy in the different genders. As our numbers continue to grow, we hope that we will be able to do so. As of now, we are only able to report trends.

Parental support has been shown to protect against mental health problems in transgender adolescents. Children who are socially transitioned at home, at school, and who use gender affirming pronouns represent those youths who are supported by their parents and caregivers. Being supported by family is associated with positive mental health outcomes [13] . Our data are somewhat limited by the fact that the majority of our participants had at least one supportive parent who was willing to facilitate medical and mental health intervention for the child and therefore may not apply to all transgender youths. In addition, regular visits with the medical team itself could influence depression and quality of life. Past studies have shown that having support from a multidisciplinary medical team – mental health provider, physician, surgeons – helped with quality of life and mental health [6].

## Conclusions

Transgender children and adolescent are a high-risk population for suicide and depression. Our preliminary results show negative associations between depression scores/suicidal ideation and endocrine intervention, while quality of life scores showed positive associations with intervention, in transgender youths over time in the US. These results align with previous work in the Netherlands and the UK.

## Data Availability

Data is not available as it would compromise confidentialty of the subjects participating.
